# Rates of Divergent Pharmacogenes in a Psychiatric Cohort of Inpatients with Depression—Arguments for Preemptive Testing

**DOI:** 10.3390/jox12040022

**Published:** 2022-10-28

**Authors:** Sibylle Christine Roll, Martina Hahn

**Affiliations:** 1 Department of Mental Health, Varisano Hospital Frankfurt Höchst GmbH, Gotenstraße 6-8, 65929 Frankfurt, Germany; 2 Department of Psychiatry, Psychosomatics and Psychotherapy, Goethe University Frankfurt—University Hospital, Heinrich-Hoffmann-Strasse 10, 60528 Frankfurt, Germany

**Keywords:** divergent phenotypes, pharmacokinetics, pre-emptive testing, pharmacogenetics, drug-gene interaction, pharmacogenes

## Abstract

Background: The international drug agencies annotate pharmacogenes for many years. Pharmacogenetic testing is thus far only established in few settings, assuming that only few patients are actually affected by drug-gene interactions. Methods: 108 hospitalized patients with major depressive disorder were genotyped for *CYP1A2*, *CYP2B6*, *CYP2C8*, *CYP2C9*, *CYP2C19*, *CYP2D6*, *CYP3A4*, *CYP3A5*, *NAT2*, *DPYD*; *VKORC1* and *TMTP*. Results: We found 583 (mean 5.4, median 5) divergent phenotypes (i.e., divergent from the common phenotypes considered normal, e.g., extensive metabolizer) in the 12 analyzed pharmacokinetic genes. The rate for at least one divergent phenotype was 100% in our cohort for CYP, but also for all 12 important pharmacogenes: patients had at least two divergent phenotypes. Compared to a large Danish cohort, CYP2C9 NM and IM status, CYP2C19 UM, CYP2D6 UM and DYPD (GAS 0, 1, 2) genotypes differed statistical significantly. For CYP2D6 and CYP2C19, 13% of the patients were normal metabolizers for both enzymes in our cohort, but this value was 27.3% in the Danish cohort, which is a highly significant difference (*p* < 0.0001). Conclusion: Divergent phenotypes in pharmacogenes are not the exception, but the rule. Patients with divergent phenotypes seem more prone for hospitalization, emphasizing the need for pre-emptive testing to avoid inefficacy and adverse drug effects in all patients.

## 1. Introduction

According to the general principles of pharmacology, the tolerability and efficacy of a drug depend on its concentration in the serum of the central cerebrospinal fluid, according to general principles of pharmacology. Drug metabolizing enzymes’ activity influences the concentration. These different levels of activity in individuals can lead to toxicity of medications and/or therapeutic failure at recommended standard drug dosages as the same dose generates a variance of drug serum concentrations in individuals. Therapeutic drug monitoring (TDM) as the gold standard for drug level optimization can only be applied later in the therapy when steady state is reached. Reimbursement of about 80 € in the ambulatory setting is not available and so it is not used in the ambulatory care setting as a routine test in Germany, but only in ambulatory care clinics. Pharmacogenetic testing (PGx) costs 90–330 € and is also not yet reimbursed, but the test has only to be conducted once in a lifetime. By knowing the PGx results, TDM can be reduced to a minimum and still reach a high drug therapy safety for the patient.

A large number of drug labels of Food and Drug Administration (FDA)-approved drugs contain warnings or information about potential adverse event risks, variable responses, drug-action mechanisms or genotype-based drug dosing. The labeling for some of the products includes specific actions to be taken based on the genotype. In total, 28 drug label annotations already exist for psychiatric drugs, only in the field of oncology do more drug label annotations exist [1]. In psychiatry, the evidence for adverse drug events for certain genotypes is high as the therapeutic range is narrow in many antipsychotics and antidepressants.

For the multiple sclerosis drug siponimod, genotyping of CYP2C9 is mandatory in Germany before starting the drug [2]. It was only recently updated to test DPYD before starting fluoruracil, capecitabine or tegafur by the drug agencies [3].

Besides these label annotations, there are guideline annotations. The most comprehensive guidelines are provided by the CPIC (Clinical Pharmacogenetic Implementation Consortium (www.cipicpgx.org, accessed on 1 September 2022)) and the DPWG (Dutch Pharmacogenetic Working Group (http://upgx.eu, accessed on 1 September 2022)). Some of these guidelines are already displaying cross tables for the activity of two pharmacogenes for one drug to give more detailed information on the dosage that can be used safely. Those cross tables exist for tricyclic antidepressants with *CYP2D6/CYP2C19* [4], mercaptopurines, azathioprine and thioguanine with *NUDT15/TPMT* [5] and warfarin with *CYP2C9/VKORC1* [6]. The guidelines also provide information on phenotypes where a particular drug should be avoided and recommends alternatives. These kinds of drug-gene-gene interactions are coming more into focus as new publication with results of panel testing are published, showing that the genotypes of all drug metabolizing enzymes have an influence on the serum level of the drug [7,8,9].

In contrast, the frequencies of drug metabolizing gene variants are not well studied in patients with a history of complicated antidepressant treatment trials. A better understanding of such frequencies and individual metabolism profiles may be important for the clinical outcome of the patients. Furthermore, patients with mental health disorders receive polypsychopharmacotherapy in up to 90% [10]. This puts the patient not only at risk for drug-drug- interactions, but also drug-drug-gene- interactions and drug-gene-gene- interactions [7].

In addition, there is growing evidence that pharmacokinetic drug-drug interactions are only clinically relevant in patients with certain genotypes, e.g., lansoprazole and fluvoxamine is only relevant in certain CYP2C19 genotypes [11], or metoprolol diphenhydramine drug- drug- interaction in patients with certain CYP2D6 phenotypes [12] and many others [13,14,15,16,17,18,19,20,21,22]. PGx results are therefore also important to predict the relevance of drug- drug interactions [23]. Nevertheless, genotyping is still not conducted in daily practice in Germany and elsewhere, even though pharmacogenomics can play an important role in predicting responders and non-responders to medications, avoiding adverse events, and optimizing drug dose [24,25,26,27,28,29,30,31,32,33,34].

The PharmGKB (Clinical Pharmacogenomic Knowledgebase (www.pharmGKB.org (accessed on 1 September 2022)) gives all current evidence on targets, the implications for a specific genotype for a drug, their evidence levels and information about the pharmacokinetic pathway of drugs, showing all drug metabolizing enzymes that are involved. Looking at those precise pharmacokinetic pathways, the published cross tables of the above-mentioned guidelines are still too narrow, e.g., amitriptyline metabolism does not only depend on the genotypes of CYP2C19 and CYP2D6, but also CYP3A4, CYP2C9 and CYP1A2, especially in higher concentrations. Similar results are found for other antipsychotics and antidepressants [24,35,36,37]. Panel testing is therefore recommended in psychiatry instead of single gene testing [37].

To the best of our knowledge, our group was one of the first psychiatric hospitals in Germany which applied PGx in patients with depression as part of the routine for a given time period. Many studies focus on only CYP2C19 and CYP2D6, but since more enzymes are involved in the metabolism of psychotropic drugs, we analyzed all genes that are involved in the individual response to pharmacotherapy.

## 2. Aim

To receive the full pharmacokinetic profile of the patients, our present retrospective analysis examines the frequencies of divergent (i.e., divergent from the common genotypes considered normal, e.g., extensive metabolizer or normal metabolizer) phenotypes in pharmacokinetic genes *CYP1A2*, *CYP2B6*, *CYP2C8*, *CYP2C9*, *CYP2C19*, *CYP2D6*, *CYP3A4*, *CYP3A5*, *NAT2*, *DPYD; VKORC1* and *TMTP*, the so called “very important pharmacogenes” as defined in Table 1. We hypothesized that there are many patients with divergent phenotypes in one of the pharmacogenes. Further, we hypothesized that patients with mental health disorders admitted for a depressive episode into a psychiatric hospital have a higher rate of divergent phenotypes of CYP enzymes compared to a large European cohort of mental health outpatients, which could show the importance of PGx testing to avoid hospital admissions.

## 3. Materials and Methods

PGx-testing was offered complimentary as a part of standard treatment to adult patients (≥18 years, *n* = 108) suffering from major depressive disorder admitted to a psychiatric hospital between November 2016 and July 2017. A clinical pharmacist spezialized in PGx testing helped with the implementation of PGx testing and ensured a correct interpretation of the PGx results. No patient has had a prior PGx test. The genetic testing panel included various pharmacodynamic and pharmacokinetic genes and alleles listed in Supplement Table S1. An EDTA blood sample was send to the laboratory for analysis. In the laboratory, the extracted DNA is quantified and normalized using an automated robotic platform and spectrometer. The normalized DNA is stored at 4 °C until the specimen is ready for analysis, which is carried out for both SNP and copy number variations by Realtime polymerase chain reaction (PCR). For each single nucleotide polymorphism (SNP), a PCR reaction is performed with the diluted DNA of the sample, the master mix and the specific assay. Each assay contains two PCR primers and two fluorescence-labeled probes. The probes are short DNA molecules that specifically bind to the sequences of the wild type or the mutant. For the determination of the copy number variants (CNVs) by a PCR reaction diluted DNA of the sample is mixed with a master mix, a reference assay (housekeeping gene with non-variable copy number) and the assay specific for analysis of CYP2D6 CNVs. The assay also contains two PCR primers, but only one probe that binds specifically to the sequence of the gene.

For our present analysis, we focused on pharmacogenes (*CYPs*, *NAT2*, *VKORC1*, *TMPT*, *DYPD*) (Table 1). The data is presented in the way that the new CPIC guideline recommends for genetic test results [38,39].

A retrospective analysis of the frequency of each phenotype was conducted and the numbers of divergent phenotypes per patient documented. We compared our cohort to a large Danish cohort with severe mental illnesses (SMI) [40] to test, if our inpatient cohort is different to the outpatient register cohort of the neighbor and therefore ethnically similar country, which would confirm our hypothesis that divergent phenotypes increase the risk for hospitalization, as proposed by Alshabeeb et al. [41]. Using the patient files, the following data was collected: patient demographics, medication on prior and after PGx- testing and pharmaceutical intervention, and genotyping results. The data were collated using Microsoft Excel 2010, Version 14.0.7194.5000 (Microsoft Corp., Redmond, WA, USA).

Statistical analysis: For the two- tailed z-test to compare our cohort to the first published large European mental health disorders outpatient cohort from northern Europe, we used science statistics calculator (https://www.socscistatistics.com/tests/ztest/default2.aspxsocial, accessed on 1 September 2022). Significance level was defined as *p* < 0.05.

The retrospective analyses received approval by on 9/27/2018 the Hesse ethics committee (approval FF88/2018).

## 4. Results

### 4.1. Demographics

In total, 46 men (43%) and 62 women (57%) were genotyped. Mean age was 44.17 ± 14.42 years.

A total of 94 patients had a chronic depression (ICD-10: F33.2 and ICD-10: F33.3), ten patients were having their first depressive episode (ICD-10: F32.2). Four patients had a bipolar depression (ICD-10: F31.4). Twenty-four patients were not taking antidepressants at the time of PGx testing, 84 patients took antidepressants and wanted to be switched to another antidepressant due to either side effects or inefficacy.

### 4.2. Phenotypes

#### 4.2.1. CYP-Enzymes (*n* = 108)

**Figure 1:**
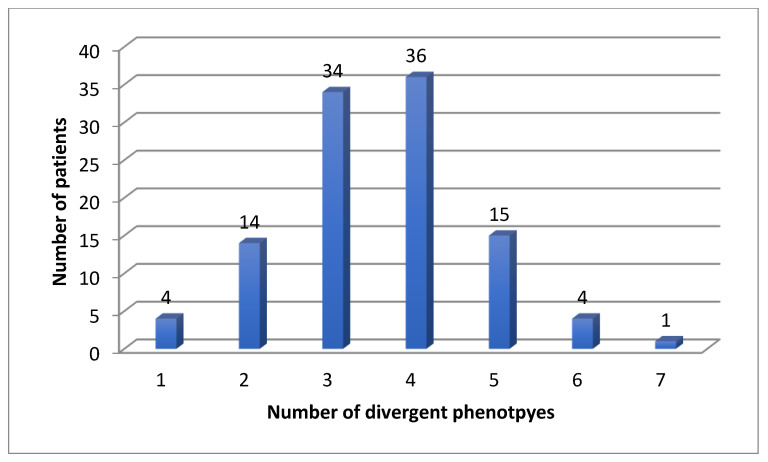
Number of divergent phenotypes for CYP enzymes in 108 patients.

Eighty-four patients were receiving antidepressants at the time of PGx testing, 24 were treatment naïve. For the CYP enzymes, we found four (4%) patients with one, 14 (13%) patients with two, 34 (31%) patients with three, 36 (33%) patients with four, 15 (14%) patients with five, four (4%) patients with six and one (1%) patient with seven divergent phenotypes (Figure 1).

In the eight CYP genes, we found a mean of 3.55 and a median of 4 divergent phenotypes.

#### 4.2.2. DPYD, NAT2, VKORC and TMTP (*n* = 108)

**Figure 2:**
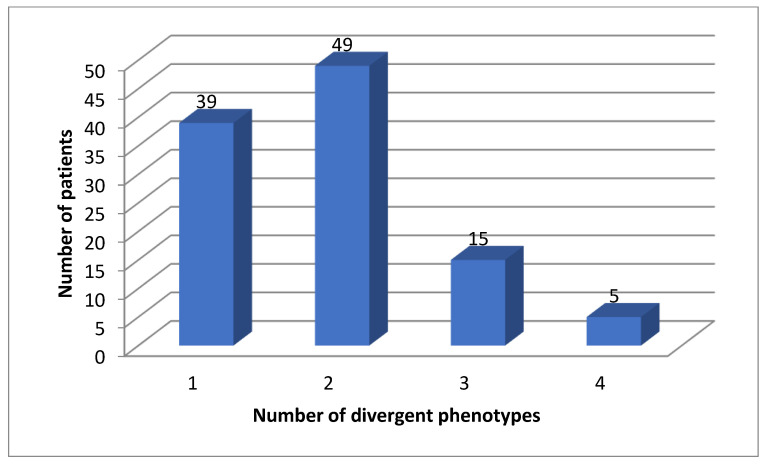
Number of actionable phenotypes for *DPYD*, *TMTP*, *NAT2* and *VKORC1*.

All patients (100%) had divergent phenotypes of either one of these genes (Figure 2).

#### 4.2.3. Very Important Pharmacogenes (*n* = 108)

**Figure 3:**
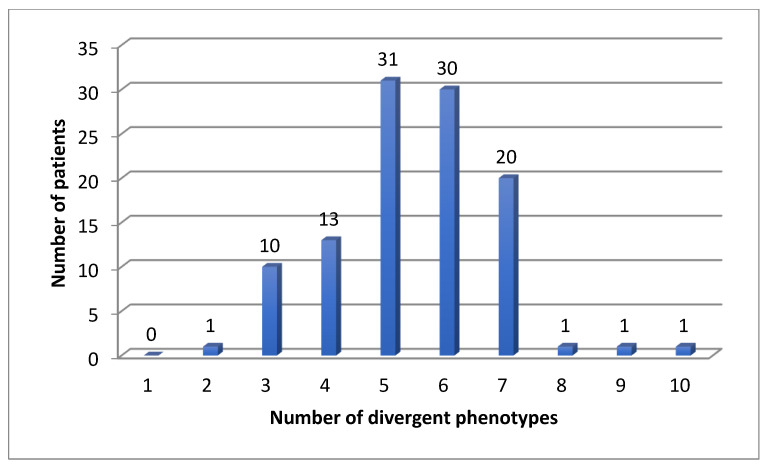
Number of divergent phenotypes in 108 patients for 12 pharmacokinetic genes.

We found 583 (mean 5.4, median 5) divergent phenotypes in the 12 analyzed pharmacokinetic genes. The rate for at least one divergent phenotype was 100% in our cohort for CYP, but also for all 12 important pharmacogenes: patients had at least two divergent phenotypes (Figure 1, Figure 2 and Figure 3). The median number was 5 (Figure 3). Three patients had more than 7 divergent phenotypes.

### 4.3. Comparison to a Danish Cohort 

We compared our results to a large Danish outpatient cohort of patients with severe mental diseases for 8 of the twelve pharmacogenes that were published from the Danish cohort [40]. Results can be found in Table 2.

## 5. Discussion

### 5.1. Frequencies of Divergent Genotypes

We found that 100% of patients had a divergent phenotype for at least one pharmacogene and a median of 5. This is comparable to other findings in a Danish cohort [40]. They used a partly different panel, but also found that 99.9% of the cohort had at least one divergent phenotype. A study from the Netherlands found that 100% of patients were receiving a drug with a metabolism that is affected by a polymorphic gene [26], so that it can be concluded that every patient might benefit from pre-emptive testing of the pharmacogenes. 

The high rate of divergent CYP-phenotypes emphasizes how important panel testing versus single gene testing is: psychotropic drugs often are metabolized by multiple CYPs, e.g., sertraline is metabolized by CYP2D6, CYP2C19, CYP3A4 and CYP2C9 [42]. All CYPs have an influence on the serum concentration of the parent drug and its active and inactive metabolites. Differences in the composition of those divergent phenotypes could explain differences in efficacy and tolerability of one drug, as we know it for amitriptyline and its metabolite nortriptyline [4]. Indeed, the “common” patient with normal metabolizer status in all pharmacogenes is really “uncommon” if you are considering the variability of CYP enzymes and pharmacodynamics genes [43]. For the 8 CYP enzymes that were tested, there are 21 phenotypes that result in over 40,000 different combinations of those 21 phenotypes. As seen in the presented data, only 13% are normal metabolizers in both CYP2D6 and CYP2C19 [44], which is even lower than in a large Danish cohort with 77,684 mental health disorder patients where 27.3% of patients were normal metabolizers for both enzymes. This also supports our hypothesis that the risk for hospitalization increases in patients with divergent phenotypes. The vast majority of individuals had one or more divergent phenotypes and might require therapy adjustments based on PGx guidelines.

The metabolic profile of the patients is very diverse, making it impossible for the psychiatrist to gain clinical experience with patients of a particular metabolic profile as the chance of seeing a patient with the same profile during his/her entire career is very unlikely. Careful individualized pharmacokinetic profile interpretation is needed. As pharmacists have a deep knowledge in pharmacokinetic mechanisms, clinical guidelines recommend consulting with a clinical pharmacist if a divergent phenotype is discovered [4]. Interprofessional collaboration between the psychiatrist and a clinical pharmacist is helpful, especially during the implementation phase of PGx testing in the hospital setting and for interpretation of PGx results [45].

### 5.2. Comparison to a Danish Cohort

Differences to the Danish cohort were especially found for *CYP2D6*, *CYP2C19*, *CYP2C9* and *DPYD*. The statistically significant differences interestingly affected ultra-rapid metabolizer status for CYP2D6 (*p* < 0.0001) and CYP2C19 (*p* < 0.00224) and normal metabolizer status for CYP2C9, which leads to a faster metabolism compared to the Danish cohort of common antidepressants like citalopram, sertraline, amitriptyline, and others. Also, for CYP2D6 *1/*4xN, were due to the analysis method of the laboratory, a genotype could not be stated, but is either ultra-rapid or intermediate metabolizer status, we found a highly significant difference (*p* < 0.00001). If those patients are accounted for IM, the *p*-value would be 0.033. If accounted for UM, the statistical significance would be below *p* < 0.00001. This statistically significant shift towards faster metabolism proofs our hypothesis that patients with inefficacy due to divergent phenotype might have a higher risk of hospitalization.

These differences could be due to the different psychiatric disorders of the cohorts: while the Danish cohort also contains bipolar, schizophrenic, autism and ADHD patients, our cohort has a depression and bipolar only. Antidepressants are prone to genotypes in comparison to stimulants and mood stabilizers. So, for CYP2D6 UM and CYP2C19 UM (and RM, but without statistical significance = 0.08) the differences can be explained by the history of antidepressant treatment in our cohort: 84 patients were taking an antidepressant on admission (AD-treated group) and prior to PGx testing. 51 patients were receiving an antidepressant with a guideline annotation, 28 actionable genotypes were found, mostly ultra-rapid metabolizer status in SSRIs. Many patients probably did not respond to the general practitioner’s or ambulatory psychiatrist’s antidepressant treatment leading to the admission to the psychiatric hospital. Recent meta-analyses with five prospective randomized-controlled trials on depressive symptom remission, showed that patients receiving pharmacogenetic-guided therapy (*n* = 887) were 1.7 and 1.74 times more likely to achieve symptom remission as compared to patients receiving usual treatment (*p* = 0.005) [33,46], which leads to the same conclusion. Therefore it seems reasonable to assume that preemptive testing could have avoided admission to the psychiatric hospital, at least in some patients. Also, as seen in the STAR*D trial, the rate of response to initial antidepressant treatment was only 49.6% [47], and a systematic review showed that non-responders to one or more treatments have a 15% likelihood of suicide ideation compared to 6% of patients with treatment-responsive depression and 1% in the general population [48]. Suicidal ideation is the cardinal symptom that is leading to admissions in a psychiatric hospital. Preemptive genotyping could reduce the number of adverse drug reactions and inefficacy, especially in psychopharmacotherapy where the efficacy of the antidepressant can only be evaluated after 2 weeks. However, prospective studies with a larger number of patients are needed to gain statistical power for this hypothesis.

That a divergent phenotype might increase the odds for an admission to the hospital can also be assumed due to the fact that the number of patients with non-normal phenotypes is higher compared to other southern European cohorts [49], and also to the northern Danish cohort (*p* > 0.0001). Statistically significant differences could be found for UM, RM, NM and IM phenotype [44]. Since Germany is between the southern and northern European countries of which there is genotype/phenotype data available and we found the same discrepancies (more ultra- rapid metabolizers), we assume that it is a selection bias of the admission to the hospital in our cohort, that leads us to the conclusion that divergent genotypes increase the risk of hospitalization.

Also, if the antidepressants are overdosed (in IM and PM), it might lead to an early discontinuation of the drug and chronicity of depressive symptoms, leading to an admission at a psychiatric hospital as shown by a study with 2066 patients, in which the CYP2C19 UMs and CYP2C19 PMs were more prone to switch escitalopram to another antidepressant [34]. Ultra rapid metabolizers were much more common in our inpatient cohort (*p* = 0.002).

Differences could also be due to other genetic backgrounds of the Danish and the German population. This also emphasizes the importance of the precision medicine approach in every drug therapy: to test before starting a drug can prevent adverse drug events and a lack of drug response [50].

The differences that were found for *DPYD* between the Danish and our German cohort cannot be explained as most of the patients probably never received 5-floururacil. However, co-medication and medication history was not available in our analysis, so the question stays unanswered. *DPYD* is not involved in metabolism of antidepressants. Prospective studies should be conducted in the future to analyze *DPYD* genotypes and their enrichments in certain cohorts or ethnicities. The relatively high number of patients with lower activity scores emphasized the importance to conduct a pre-emptive testing before starting 5-fluruacil, as recommended by the drug agencies [3].

## 6. Limitations

The study was conducted as a retrospective analysis of naturalistic data. The sample size is small, increasing the type 2 error. Co-medication was not documented so that phenoconversion effects are unknown and might have altered the number of patients with actionable genotypes due to gene-drug interactions [7]. Phenoconversions lead to a much higher rate of actionable genotypes as shown by Moustafa et al. 95% of patients had an actionable genotype [51]. In our cohort (without phenoconversion effects) only 51% of the patients had actionable genotypes [52].

Selection bias might have altered the results: even if PGx- testing was offered to all patient with major depressive disorders during this period of time, we do not know how many refused the offer.

Pharmacodynamic genes like *5HTR2A*, *OPRM1*, *COMT* and others might also play an important role in side effects, relevance of pharmacodynamics drug-drug interactions and efficacy of the antidepressant. However, this is still discussed controversial, while pharmacokinetic genes have reached guideline status for many years already. Their influence on hospitalization needs to be analyzed in the future to gain more understanding of the role of all genes on efficacy and adverse drug reactions to psychotropic drugs. Results from the PRIME Trial (pharmacogenes only) in comparison to the GUIDED Trial (pharmacodynamic and pharmacokinetic genes) are not differing substantially in remission and response rates in patients with major depressive disorder, questioning the impacts of pharmacodynamic genes on patient outcomes [53,54]. The “uncommon” metabolism profile is very common as 100% of our cohort had divergent genotypes. Genetic testing can reduce the length of stay, prevent rehospitalizations and improve patient outcomes (GAF score and CGI score) [49,55]. As the role of clinical pharmacists is evolving, collaborative care models, including clinical pharmacists’ services (available in the U.S. and Germany) are needed [56]. Ignoring the scientific evidence on the influence of genetic polymorphism on the pharmacokinetics of antidepressants and antipsychotics put patients at risk of adverse drug reactions or inefficacy of the drugs that might lead to chronicity of the disease and/or admission to a psychiatric hospital [53]. Further, pharmacogenes are important for 70–80% of all clinically used drugs and therefore PGx plays an important role in all patients who require a drug therapy, regardless of ethnicity [57].

## 7. Further Studies

New RCTs like the “PRIME Care” trial are on the way to increase the scientific evidence on cost effectiveness of preemptive testing in mental health [52]. If cost-effectiveness can be shown and reimbursement is achieved, PGx testing could be used more widely and more studies with larger cohorts could increase the knowledge on the relevance of pharmacogenes on remission and response rates. This could result in a better efficacy and drug safety for the patient in the near future.

## 8. Conclusions

Every patient had at least one divergent phenotype of one important pharmacogene. This and the high number of divergent phenotypes for CYP2D6 and CYP2C19 emphasizes the importance of pre-emptive panel testing versus reactive single-gene testing in psychiatry, since most of the PGx-guidelines give recommendation for the *starting* dose of the antidepressant and antipsychotic. As the number of patients with depressive disorders is increasing, the implementation of PGx is not only of benefit for the patient, but for the health care system and the economy. Larger randomized controlled trials are needed to confirm our findings. Clinical pharmacists on the ward should be involved before starting PGx testing as they can assist the implementation and interpretation of the PGx results. Health system regulators should consider reimbursement in the near future to achieve a higher drug therapy safety for all patients.

## Figures and Tables

**Table 1:** Definition of divergent phenotypes for the analysis of important pharmacogenes. GAS = gene activity score; NM = normal metabolizer, IM = intermediate metabolizer, RM = rapid metabolizer, PM = poor metabolizer, UM = ultra-rapid metabolizer; * = allele.

Gene	Variants and Alleles That Were Counted as Divergent
*CYP1A2*	Increased inducibility or decreased function (*1F, *1C)
*CYP2B6*	PM, IM (*2, *18, *6)
*CYP2C8*	Reduced activity (*3)
*CYP2C9*	Decreased function and no function (*2, *3, *6, *8, *11)
*CYP2C19*	PM, IM, RM, UM, (*2, *3, *4, *17)
*CYP2D6*	Activity score below 1.25: PM, IM, UM
*CYP3A4*	Increased activity (*1B, *1G)
*CYP3A5*	NM (*1), as PM is the wild type in Caucasians (90%)
*DPYD*	PM, IM
*NAT2*	Rapid, Slow (*5B, *6A, *7B)
*TMTP*	PM, IM (homo- and heterozygous)
*VKORC1*	NM/IM, IM/IM (homo- and heterozygous carriers)

**Table 2:** Frequencies of a German MDD cohort in comparison to a large Danish cohort with SMD, the z-values and *p*-values of the analysis. Statistically significant differences are highlighted. GAS = gene activity score; NM = normal metabolizer, IM = intermediate metabolizer, RM = rapid metabolizer, PM = poor metabolizer, UM = ultra-rapid metabolizer.

Gene/Variant	German Cohort with MDD (*n* = 108)	Danish Cohort with SMI (*n* = 51,464)	z-Value	*p*-Value
*CYP3A5*				
Homozygous	19.4%	0.6%	−1.8414	0.066
Heterozygous		12.8%		
Non-Expressor	80.6%	86.6%	1.8548	0.064
*CYP2B6*				
NM	60%	58,4%	−0.3788	0.70394
IM and PM	40%	41,6%	0.3788	0.70394
*CYP2C9*				
**NM**	**83%**	**66.3%**	**−3.7519**	**0.0018**
**IM**	**17%**	**30.2%**	**3.2703**	**0.00108**
PM	1%	3.6%	1.474	0.14156
*CYP2C19*				
**UM**	**9%**	**3.7%**	**−3.0349**	**0.00244**
RM	31%	25.8%	−1.1296	0.25848
**NM**	**32%**	**43.5%**	**2.3238**	**0.00203**
IM	27%	24.8%	−0.4942	0.62141
PM	2%	2.1%	0.236	0.81034
CYP2D6				
**UM**	**1%**	**0%**	**21.8295**	**<0.00001**
NM	54%	62.4%	1.8564	0.06288
IM	35%	33.5%	−0.3743	0.71138
PM	7%	4.2%	−1.6953	0.08914
**UM/PM (**(*1/*4)xN)	**3%**	**0%**	**−37.8106**	**<0.00001**
*DPYD*				
**GAS 2**	**76%**	**98%**	**16.1002**	**<0.00001**
GAS 1.5	2%	0.1%	−1.4595	0.1443
**GAS 1**	**19%**	**<1.4%**	**−16.118**	**<0.00001**
GAS 0.5	0%	<0.02%	0.1024	0.92034
**GAS 0**	**3%**	**<0.02%**	**−23.074**	**<0.00001**
*VKORC*				
Heterozygous	38.9%	37.2%	−0.303	0.718
Homozygous	13.8%	15.3%	0.4046	0.682
*TMPT*				
NM	94.4%	90.2%	1.4824	0.139
IM	4.6%	9.6%	1.7527	0.080
PM	0.9%	0.3%	1.1885	0.234

For CYP2D6 and CYP2C19, 13% of the patients were normal metabolizers (NM) for both enzymes in our cohort, but 27.3% in the Danish cohort, which is a highly significant difference (*p* < 0.0001). For *DPYD*, *TMTP*, *NAT2* and *VKORC1* all patients had at least one divergent genotype (Figure 3).

## Data Availability

The raw data is reported in Supplement Table S1. Additional data can be retrieved from martina.hahn@varisano.de.

## Supplementary Materials

The following supporting information can be downloaded at: https://www.mdpi.com/article/10.3390/jox12040022/s1, Table S1: phenotype raw data of all patients.
